# “Snowball” in the Treatment of Alcohol Use Disorder

**DOI:** 10.1192/j.eurpsy.2025.434

**Published:** 2025-08-26

**Authors:** H. O. Torun, B. Akdöner, T. Sarıkavak

**Affiliations:** 1 Psychiatry, İzmir City Hospital, İzmir; 2 Psychiatry, Near East University, Girne; 3 Psychiatry, Atlas University, İstanbul, Türkiye

## Abstract

**Introduction:**

Treatment motivation is very important in the treatment of alcohol and substance use disorders. The evaluation of motivation, which is seen as the first step in addiction treatment, will also help understand the person’s interest and compliance with treatment. The lack of motivation of the person during the treatment process causes treatment abandonment and relapses.

An important issue in the treatment of alcohol use disorder is the trust of the person in the treatment. It is also important for the addict to have someone around him who has benefited from treatment, which is very important in terms of treatment compliance. With the influence of a person who has benefited from addiction treatment, the likelihood of applying for treatment and benefiting from treatment increases in a “snowball” manner.

**Objectives:**

The aim of this study was to evaluate whether people who are treated for alcohol use disorder have a treatment motivation for those around them. It was aimed to examine whether the people who have been treated are important in increasing the trust in treatment for other patients.

**Methods:**

The study included cases who applied to the Seferihisar State Hospital Psychiatry Outpatient Clinic with complaints of alcohol use. The sociodemographic information form was filled out during the individuals’ first applications. The participants were asked whether there were people around them who had received inpatient treatment for alcohol use disorder at the hospital where the study was conducted, and the individuals were then administered the Readiness for Change and Desire for Treatment Scale (SOCRATES), Treatment Motivation Questionnaire (TMQ), and Alcohol Use Disorders Identification Test (AUDIT). The individuals were given the standard treatment recommended in our country’s treatment guidelines for the diagnosis of alcohol use disorder. The individuals participating in the study were interviewed at 1 month, 2 months and 3 months after starting treatment. The individuals’ answers and the decrease in the frequency and amount of alcohol use were recorded.

**Results:**

The rate of treatment attendance is 2.7 times higher for people who have received treatment for alcohol use disorder in their circle.

The rate of treatment confidence in treatment is 4.1 times higher for people who have received treatment for alcohol use disorder in their circle.

The frequency and amount of alcohol use has decreased more for people who have received treatment for alcohol use disorder in their circle.

**Conclusions:**

It has been determined that those who are surrounded by people who have received treatment for alcohol use disorder have higher levels of treatment confidence and compliance. The assistance of these people to those around them can play an important role in increasing the success of addiction treatment.

**Disclosure of Interest:**

None Declared

